# Supplementary material for: Single-Walled Carbon Nanotube Synthesis Yield Variation in a Horizontal Chemical Vapor Deposition Reactor
Source: Nanomaterials (Basel). 2021 Dec 4;11(12):3293. doi: 10.3390/nano11123293 (PMC8706368; doi:10.3390/nano11123293)
Supplement: Supplementary file 1 [file nanomaterials-11-03293-s001.zip › nanomaterials-1451766-supplementary.pdf]

Supporting Information

Single-walled carbon nanotube synthesis yield variation in a horizontal chemical vapor deposition reactor

Sung-Il Jo and Goo-Hwan Jeong *

Department of Advanced Materials Science and Engineering, Kangwon National University, Chuncheon, Gangwon-do 24341, Republic of Korea; sungil107@kangwon.ac.kr (S.-I.J.); * ghjeong@kangwon.ac.kr (G.-H.J.); Tel.: +82-33-250-6268

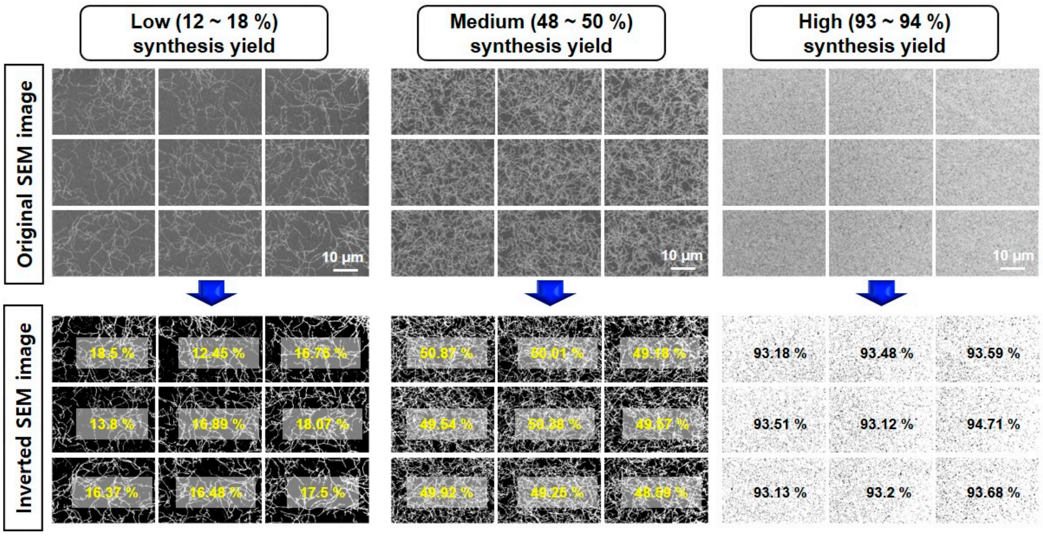

Figure S1. Detailed examples of low, medium, and high synthesis yields of SWNTs.

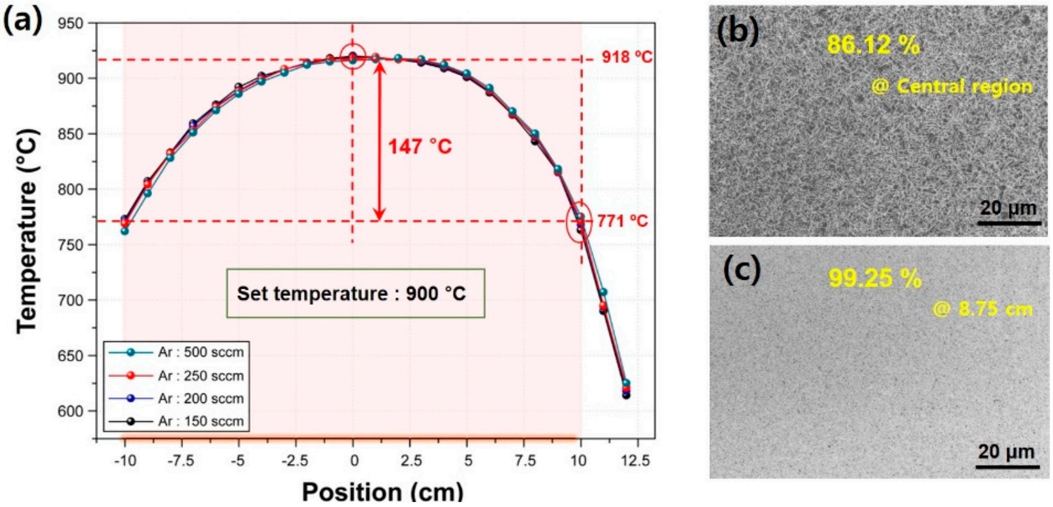

Figure S2. (a) Temperature profile of the inner space of the CVD chamber. SEM images showing SWNTs grown at (b) center and (c) rear edge region.

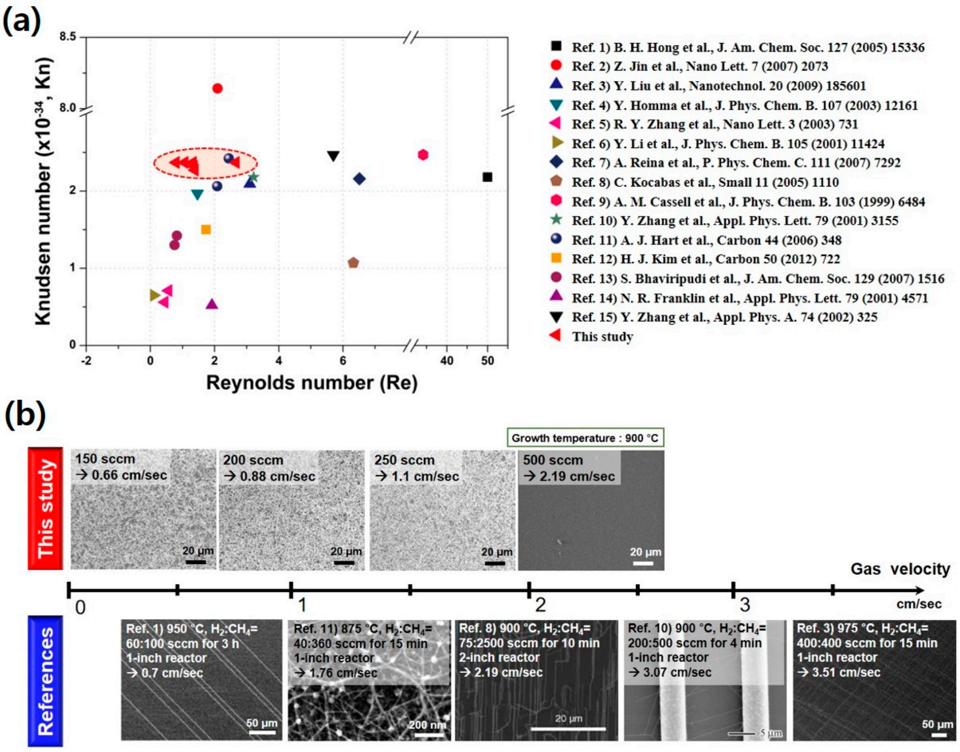

Figure S3. Comparison of (a) the Reynolds and Knudsen numbers and (b) gas velocity between this study and previous studies.

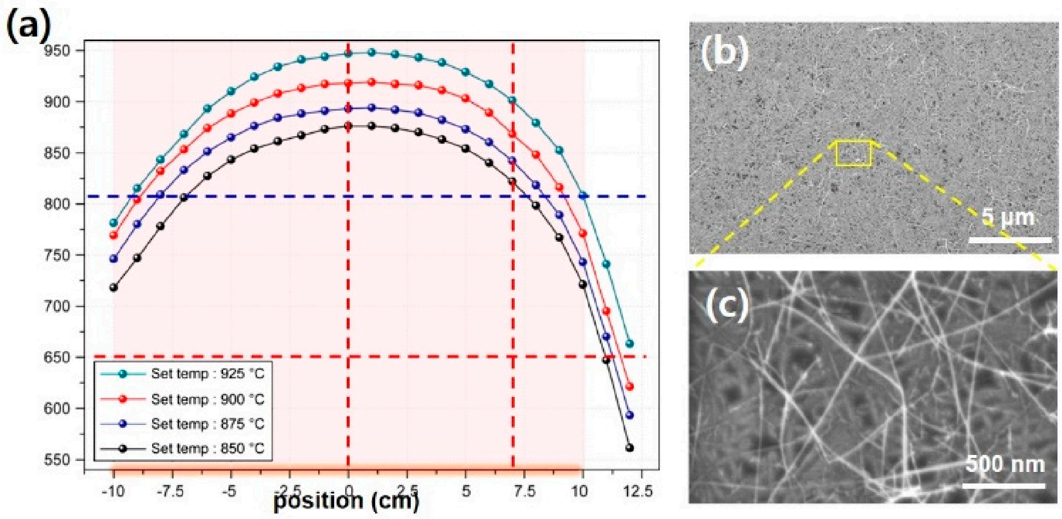

Figure S4. (a) Temperature profile of the inner space of the CVD chamber at different set temperatures. (b) and (c) SEM images showing high density SWNTs grown at the furnace edge region at 925 °C.

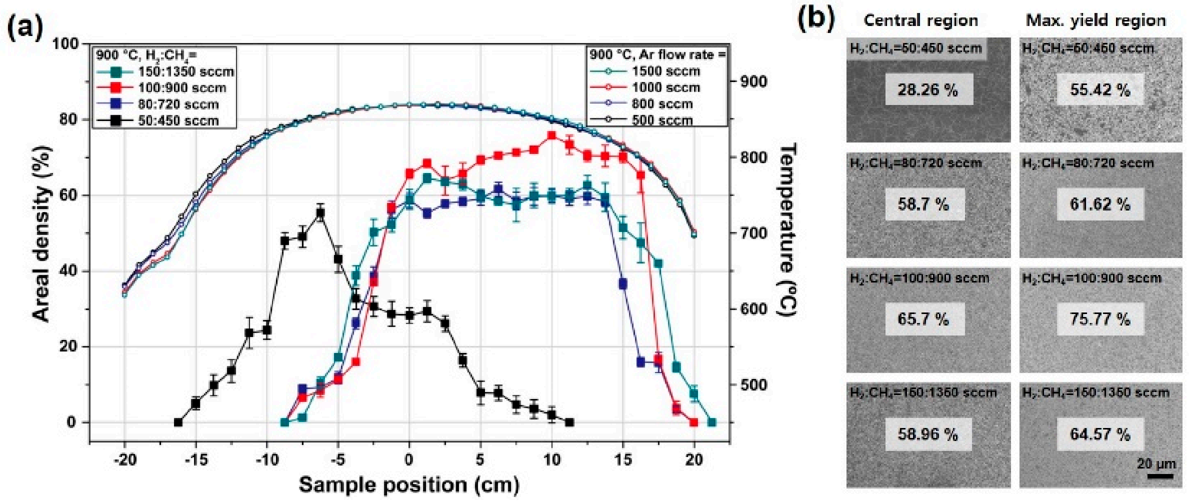

Figure S5. (a) Variation of the areal density of SWNTs with respect to the gas flow rate and sample position in the 2-inch diameter CVD chamber. Right axis shows temperature distribution along the CVD furnace at specified set temperature. (b) HRSEM images show areal density and SWNTs grown at central and maximum-growth-yield regions using different gas flow rate.

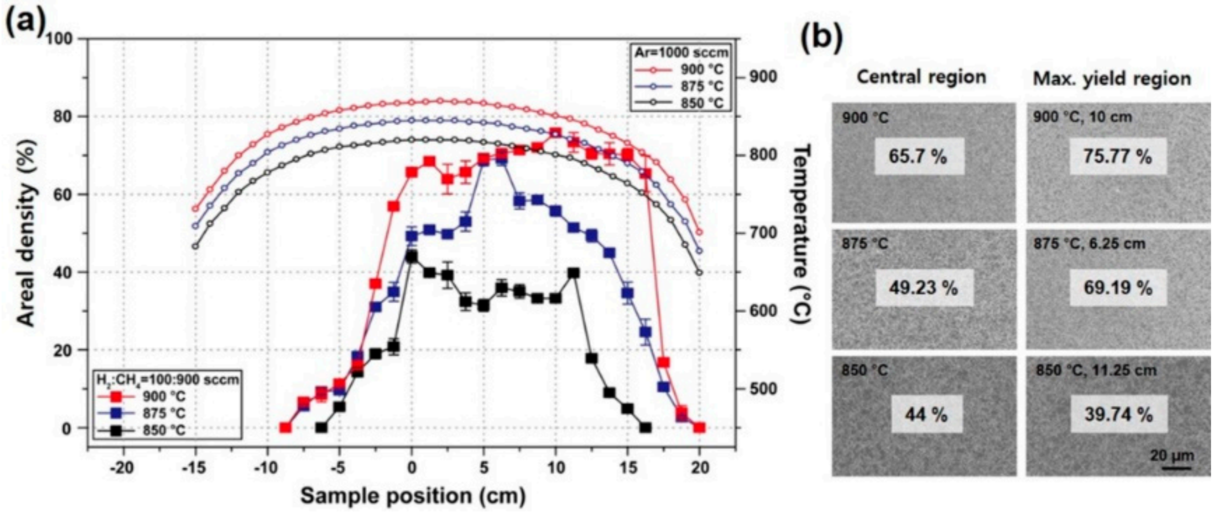

Figure S6. (a) Variation of the areal density of SWNTs with respect to the growth temperature and sample position in the 2-inch diameter CVD chamber. Right axis shows temperature distribution along the CVD furnace at specified set temperature. (b) HRSEM images show areal density and SWNTs grown at central and maximum growth yield regions using different growth temperature.

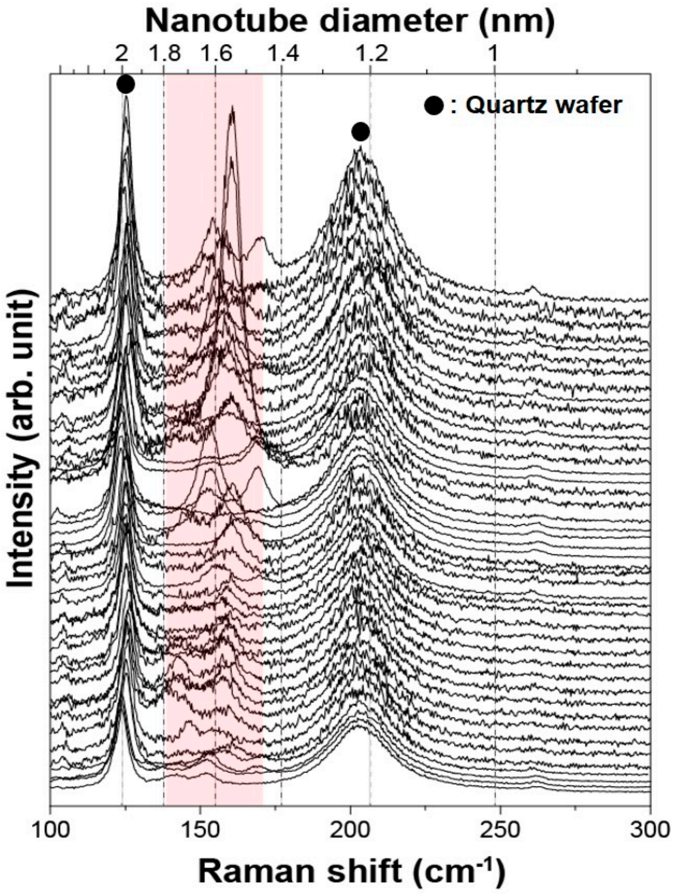

Figure S7 Raman spectra for HA-SWNTs grown on the quartz wafer installed in the maximum-synthesis-yield region. Regions where RBM peaks were detected are denoted by color block.
